# Supplementary material for: UBE3A and transsynaptic complex NRXN1-CBLN1-GluD1 in a hypothalamic VMHvl-arcuate feedback circuit regulates aggression
Source: bioRxiv. 2023 Mar 1:2023.02.28.530462. Preprint. [Version 1] doi: 10.1101/2023.02.28.530462 (PMC10002692; doi:10.1101/2023.02.28.530462)
Supplement: Supplement 1 [file NIHPP2023.02.28.530462v1-supplement-1.pdf]

bioRxiv preprint doi: https://doi.org/10.1101/2023.02.28.530462; this version posted March 1, 2023. The copyright holder for this preprint (which was not certified by peer review) is the author/funder, who has granted bioRxiv a license to display the preprint in perpetuity. It is made available under aCC-BY 4.0 International license.

SUPPLEMENTARY FIGURES

figure S1

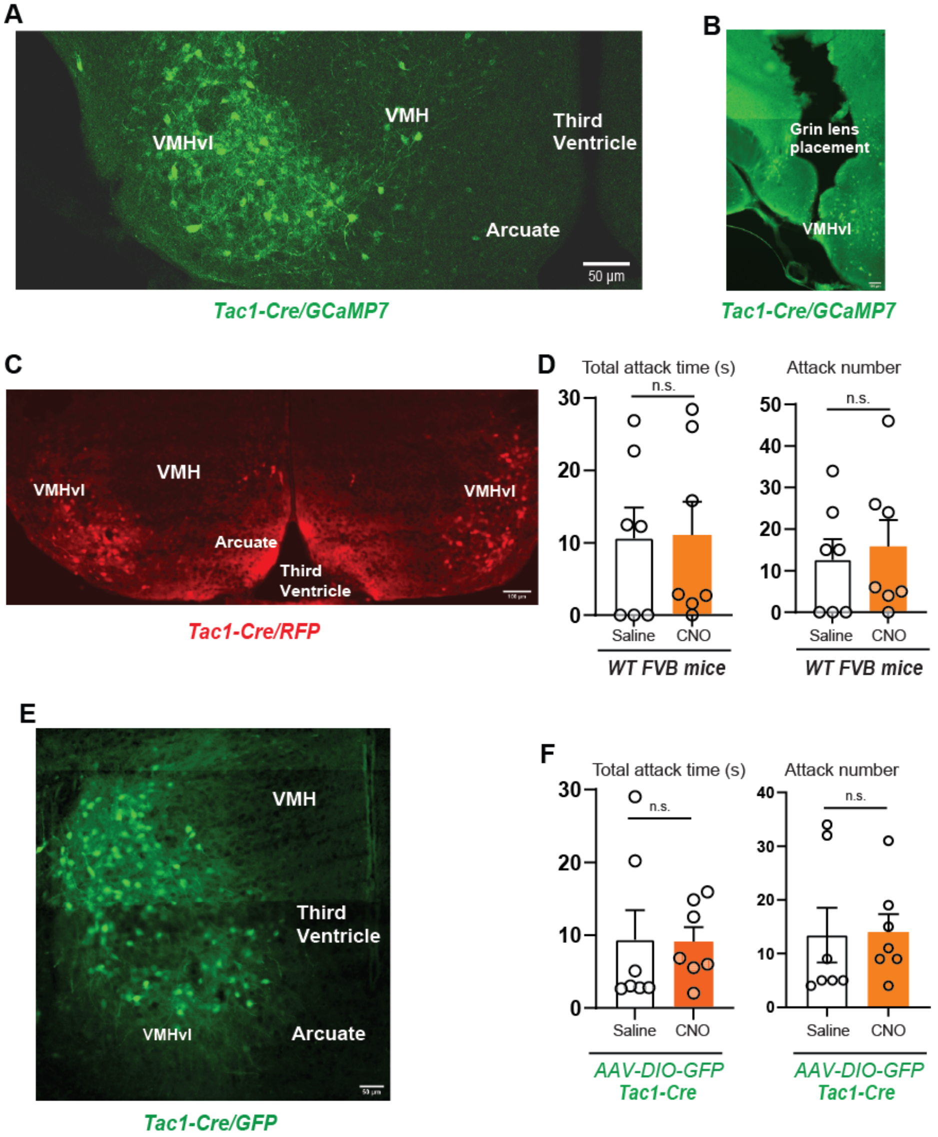

fig. S1 VMHvl *Tac1* neurons drive attack behavior.

(**A**) Representative image of *GCaMP7f* expression in VMHvl *Tac1* neurons after stereotactic injection of AAV9-hSyn-DIO-GCaMP7f virus into VMHvl of *Tac1-Cre* male mice (scale bar, 50 µm). (**B**) Representative image of Grin lens placement in VMHvl of AAV9-hSyn-DIO-GCaMP7f injected *Tac1-Cre* male mice (scale bar, 100 µm). (**C**) Representative image of anti-DsRed antibody immunofluorescence staining in *Tac1-Cre* mice injected with AAV-DIO-

bioRxiv preprint doi: https://doi.org/10.1101/2023.02.28.530462; this version posted March 1, 2023. The copyright holder for this preprint (which was not certified by peer review) is the author/funder, who has granted bioRxiv a license to display the preprint in perpetuity. It is made available under aCC-BY 4.0 International license.

hM3D(Gq)-RFP in VMHvl (scale bar, 100 µm). (**D**) Total attack time/number in wild-type *FVB* mice comparing application of saline with CNO (1 mg/kg i.p., $n = 7$, $P_T = 0.9429$, $P_N = 0.6924$). (**E**) Representative image of *GFP* expression in VMHvl *Tac1* neurons after stereotactic injection of AAV-DIO-GFP virus into VMHvl of *Tac1-Cre* male mice (scale bar, 50 µm). (**F**) Total attack time/number in *Tac1-Cre* male mice comparing application of saline with CNO (1 mg/kg i.p., $n = 7$, $P_T = 0.9564$, $P_N = 0.9269$). $P < 0.05$ was considered statistically significant with *ns* indicating non-significant, *P <0.05, **P < 0.01, ***P <0.001 and ****P <0.0001.

bioRxiv preprint doi: https://doi.org/10.1101/2023.02.28.530462; this version posted March 1, 2023. The copyright holder for this preprint (which was not certified by peer review) is the author/funder, who has granted bioRxiv a license to display the preprint in perpetuity. It is made available under aCC-BY 4.0 International license.

figure S2

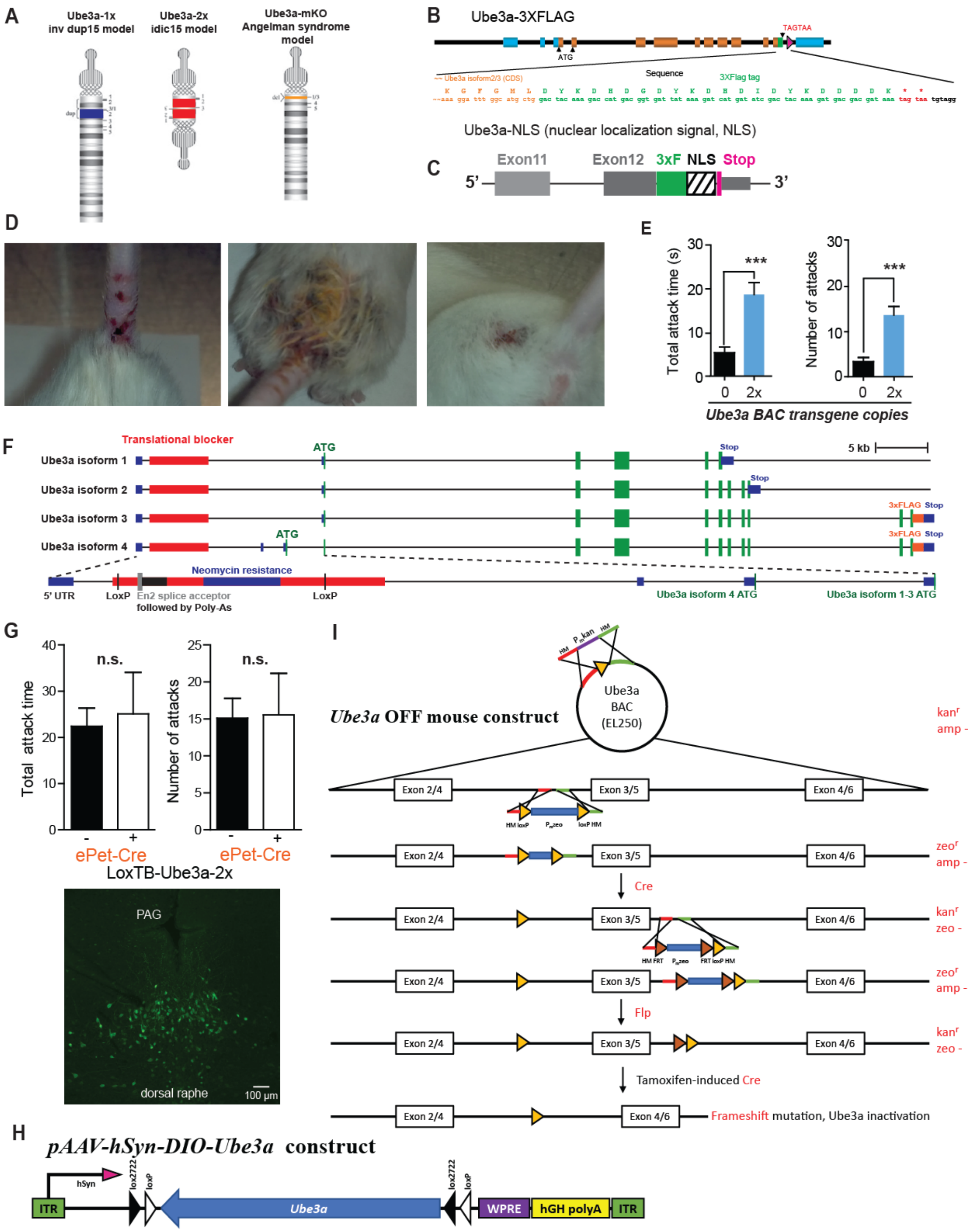

fig. S2 ***Ube3a*** **genetic and molecular constructs.**

(A) Diagram of chromosome abnormality of maternal 15q11-13 interstitial duplication, maternal extranumerary isodicentric chromosome 15 (idic 15) and Angelman syndrome. **(B)** Diagram of

bioRxiv preprint doi: https://doi.org/10.1101/2023.02.28.530462; this version posted March 1, 2023. The copyright holder for this preprint (which was not certified by peer review) is the author/funder, who has granted bioRxiv a license to display the preprint in perpetuity. It is made available under aCC-BY 4.0 International license.

construct of *Ube3a* 3xFlag transgenic mice with extra gene copies of full-length *Ube3a* gene. (**C**) Diagram of Ube3aNLS with 3xFLAG and nuclear localization signal (NLS) followed by a STOP codon added in frame to exon 12 of mouse Ube3a gene. (**D**) Photos of injured mice from cages with *Ube3a-2x* male mice. (**E**) Total attack time/number in wild type (n =23) and *Ube3a-2x* mice (n = 9, P_T = 0.0007 and P_N = 0.0003). (**F**) Diagram of *LoxTB-Ube3a* construct with transcriptional/translational stop cassette (red) containing an *En2* splice acceptor (grey) followed by polyA tails (black), all flanked by *LoxP* sites and inserted into intron 1 of full-length FLAG-tagged *Ube3a* gene designed to block all potential splice isoforms. (**G**) Upper panel: total attack time/number in *LoxTB-Ube3a-2x* mice (n = 20) comparing to *ePet-Cre* (Tg(Fev-cre)1Esd):*LoxTB-Ube3a-2x* mice (n = 9) (P_T = 0.7520, P_N = 0.9340). Lower panel: FLAG immunofluorescence in dorsal raphe, scale bar 100 μm. (**H**) Diagram of construct of *AAV-hSyn-DIO-Ube3a* that expresses *Ube3a* in a Cre-dependent manner. (**I**) Diagram of construct of *Ube3a*OFF#5, a conditional *Ube3a* transgene where *LoxP* site flank exons of the full-length untagged *Ube3a* gene. P < 0.05 was considered statistically significant with *ns* indicating non-significant, *P <0.05, **P < 0.01, ***P <0.001 and ****P <0.0001.

bioRxiv preprint doi: https://doi.org/10.1101/2023.02.28.530462; this version posted March 1, 2023. The copyright holder for this preprint (which was not certified by peer review) is the author/funder, who has granted bioRxiv a license to display the preprint in perpetuity. It is made available under aCC-BY 4.0 International license.

figure S3

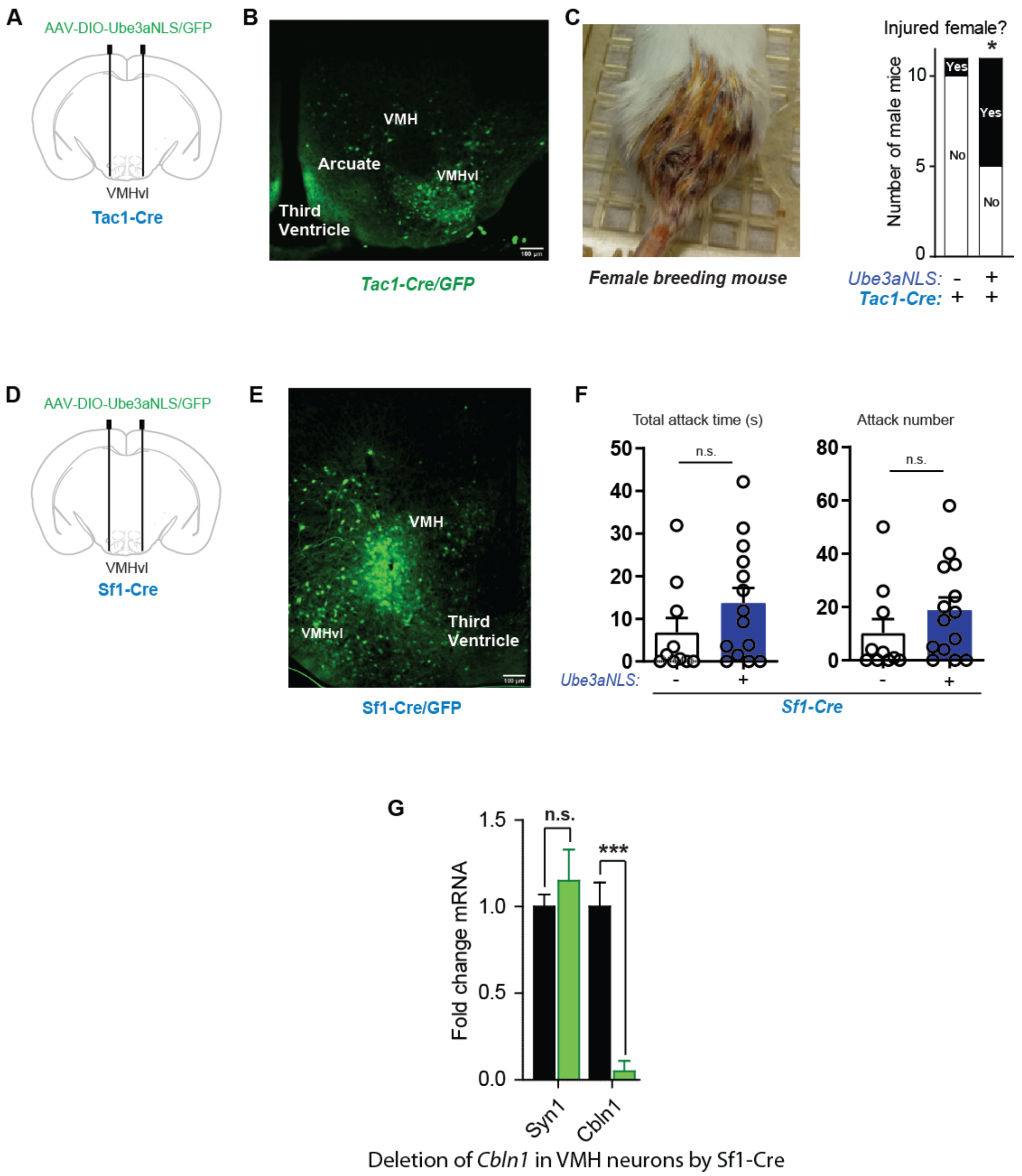

fig. S3 Aggression is increased by nuclear-targeted UBE3A expression in VMHvl *Tac1-Cre* neurons but not in VMHdm of *Sf1-Cre* neurons.

(**A**) Diagram of stereotaxic injection. (**B**) Representative image of *GFP* expression in VMHvl *Tac1* neurons when stereotactically injecting AAV-DIO-Ube3a-NLS + AAV-DIO-GFP virus into VMHvl of *Tac1-Cre* male mice (scale bar, 100 μm). (**C**) Left: representative photo of female mouse with hind injury. Right: number of male mice that injured female added to their cages in *Tac1-Cre* mice injected with AAV-DIO-GFP + AAV-DIO-Ube3aNLS (n = 11 mice) compared

bioRxiv preprint doi: https://doi.org/10.1101/2023.02.28.530462; this version posted March 1, 2023. The copyright holder for this preprint (which was not certified by peer review) is the author/funder, who has granted bioRxiv a license to display the preprint in perpetuity. It is made available under aCC-BY 4.0 International license.

to AAV-DIO-GFP (*n* = 11 mice) in VMHvl (*P* = 0.032). (**D**) Diagram of stereotaxic injection. (**E**) Representative image of *GFP* expression in VMH *Sf1* neurons when stereotactically injecting AAV-DIO-Ube3a-NLS+ AAV-DIO-GFP virus into VMHdm of *Sf1-Cre* male mice (scale bar, 100 µm). (**F**) Total attack time/number in *Sf1-Cre* male mice compared stereotactically injecting AAV-DIO-Ube3a-NLS+ AAV-DIO-GFP virus (*n* = 14) into VMHdm of *Sf1-Cre* male mice with only AAV-DIO-GFP (*n* = 10, P_T= 0.2002, P_N= 0.2460). (**G**) Combined data of quantitative RT-qPCR of *Cbln1*and *Syn1* mRNA from single neurons isolated from VMHvl in *Sf1-Cre*:$Cbln1^{flx/flx}$ (*n* = 24 neurons, green bar) comparing to $Cbln1^{flx/flx}$ (*n* = 28 neurons, black bar) (*P* = 0.0003). *P* < 0.05 was considered statistically significant with *ns* indicating non-significant, **P* <0.05, ***P* < 0.01, ****P* <0.001 and *****P* <0.0001.

bioRxiv preprint doi: https://doi.org/10.1101/2023.02.28.530462; this version posted March 1, 2023. The copyright holder for this preprint (which was not certified by peer review) is the author/funder, who has granted bioRxiv a license to display the preprint in perpetuity. It is made available under aCC-BY 4.0 International license.

figure S4

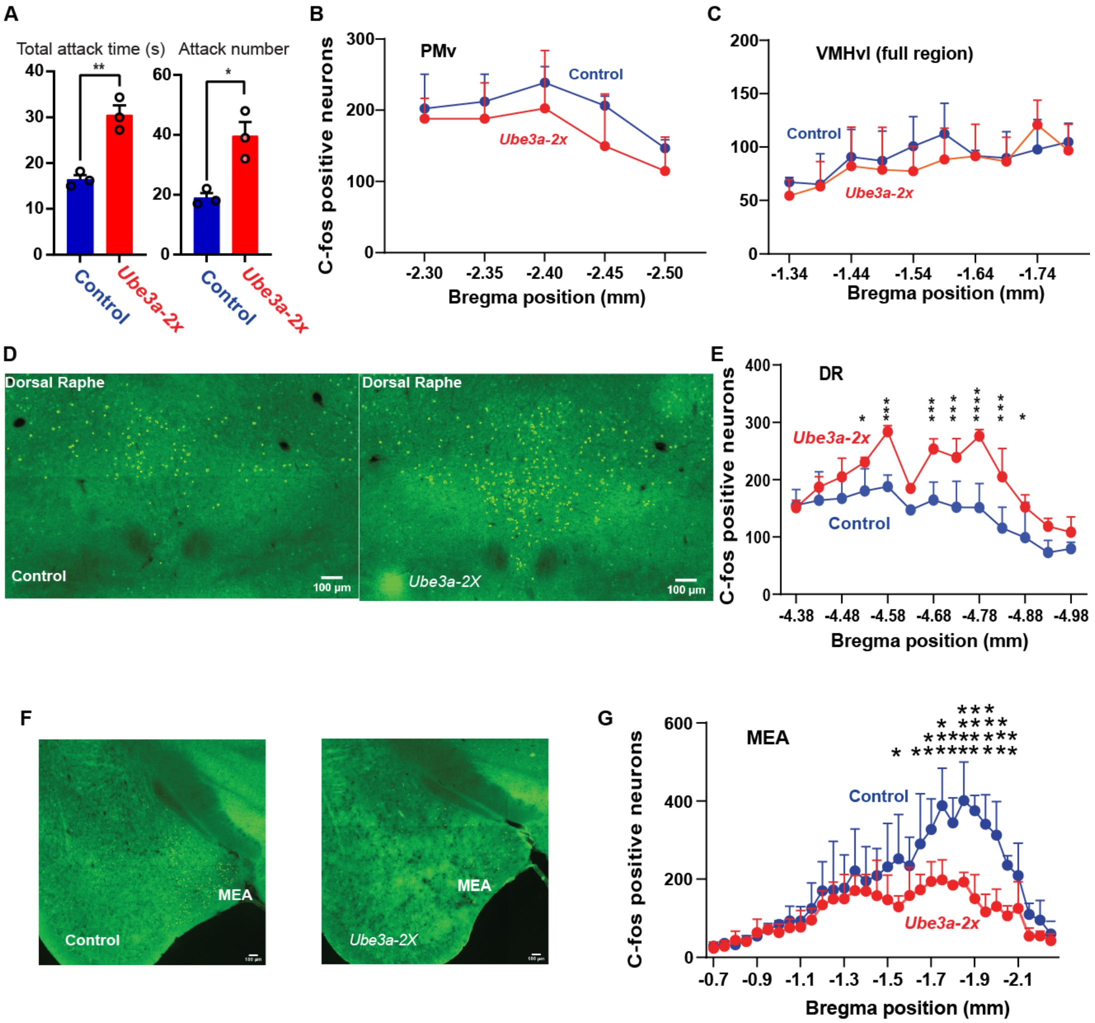

fig. S4 Images of anti-c-fos staining and quantification of c-fos positive neurons after aggression behavior test comparing *Ube3a-2x* with littermate control mice.

(**A**) Total attack time/number in aggression behavior test compared *Ube3a-2x* (n = 3) and littermate control mice (n = 3, $P_T = 0.0035$, $P_N = 0.0133$). (**B**) and (**C**) Counts of c-fos positive neurons in ventral premamillary (PMv) (**B**, $P = 0.3731$) and full VMHvl (**C**, $P = 0.3167$) after aggression behavior test compared *Ube3a-2x* compared to control littermate mice. (**D**) Representative image of anti-c-fos antibody staining in dorsal raphe compared *Ube3a-2x* and control littermate mice (scale bar, 100 microns). (**E**) Counts of c-fos positive neurons in dorsal raphe ($P = 0.0217$) after aggression behavior testing comparing *Ube3a-2x* and control littermate

bioRxiv preprint doi: https://doi.org/10.1101/2023.02.28.530462; this version posted March 1, 2023. The copyright holder for this preprint (which was not certified by peer review) is the author/funder, who has granted bioRxiv a license to display the preprint in perpetuity. It is made available under aCC-BY 4.0 International license.

mice. (**F**) Representative image of anti-c-fos antibody staining in MEA compared *Ube3a-2x* and control littermate mice (scale bar, 100 microns). (**G**) Counts of c-fos positive neurons in MEA (P = 0.1150) after aggression behavior test. All c-fos studies compared male *Ube3a-2x* (n = 3 mice) and control littermate mice (n = 3 mice). $P < 0.05$ was considered statistically significant with *ns* indicating non-significant, *P <0.05, **P < 0.01, ***P <0.001 and ****P <0.0001.

figure S5

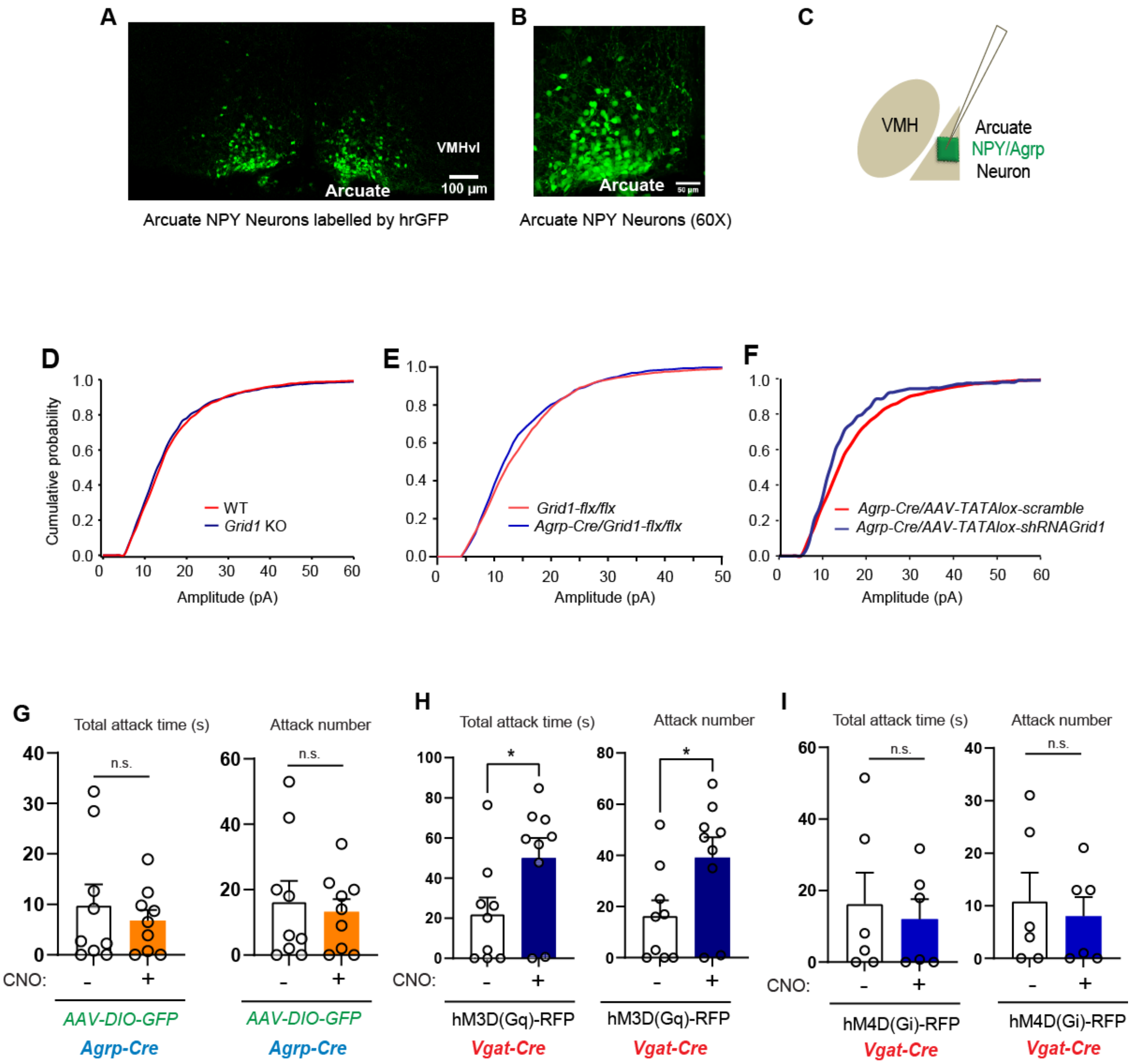

bioRxiv preprint doi: https://doi.org/10.1101/2023.02.28.530462; this version posted March 1, 2023. The copyright holder for this preprint (which was not certified by peer review) is the author/funder, who has granted bioRxiv a license to display the preprint in perpetuity. It is made available under aCC-BY 4.0 International license.

fig. S5 Miniature excitatory postsynaptic current (mEPSC) amplitude in arcuate NPY-GFP neurons when with *Grid1* loss of in arcuate AgRP/NPY neurons. (**A**) Representative image of GFP^{+} arcuate NPY neurons (labeled by *Npy-hrGFP* allele, scale bar, 100 microns) where whole–cell patch clamp recordings in arcuate were performed. (**B**) Higher magnification (60x) of GFP^{+} arcuate NPY neurons (scale bar, 50 microns). (**C**) Diagram of whole–cell patch-clamp recordings performed in GFP-labeled arcuate NPY/AgRP neurons. (**D**) Cumulative frequency plot of mEPSC amplitude in arcuate NPY GFP^{+} neurons in mice with homozygous deletion of *Grid1* (n = 4 mice, 16 cells) and control mice (n = 5 mice, 14 cells, KS test, $P > 0.05$). (**E**) Cumulative mEPSC amplitude in arcuate NPY GFP^{+} neurons in mice with *Grid1* knockout in arcuate AgRP/NPY neurons (*Grid1*-flx:flx/Agrp-Cre; n = 6 mice, 11 neurons) compared to control wild type mice (n = 4 mice, 14 cells, KS test, $P > 0.05$). (**F**) Cumulative mEPSC amplitude in arcuate NPY GFP^{+} neurons in mice with knockdown of *Grid1* in arcuate AgRP/NPY neurons (AAV-TATAlox-shRNA-Grid1 in Agrp-Cre; n = 3 mice, 6 neurons) compared to control wild type mice (n = 6 mice, 18 cells, KS test, $P < 0.001$). (**G**) Total attack time/number in *Agrp-Cre* mice (n = 9) injected with AAV-DIO-GFP comparing saline and CNO (1 mg/kg i.p., P_T = 0.5232, P_N = 0.6950). (**H**) Total attack time/number in *Vgat-Cre* mice (n =9) injected with AAV-DIO-hM4D(Gq)-RFP in the VMHvl comparing saline and CNO (1 mg/kg i.p., P_T = 0.0477, P_N = 0.0371). (**I**) Total attack time/number in *Vgat-Cre* mice (n = 6) injected with AAV-DIO-hM4D(Gq)-RFP in the VMHvl comparing saline and CNO (1 mg/kg i.p., P_T = 0.6965, P_N = 0.6738). $P < 0.05$ was considered statistically significant with *ns* indicating non-significant, *P <0.05, **P < 0.01, ***P <0.001 and ****P <0.0001.

bioRxiv preprint doi: https://doi.org/10.1101/2023.02.28.530462; this version posted March 1, 2023. The copyright holder for this preprint (which was not certified by peer review) is the author/funder, who has granted bioRxiv a license to display the preprint in perpetuity. It is made available under aCC-BY 4.0 International license.

figure S6

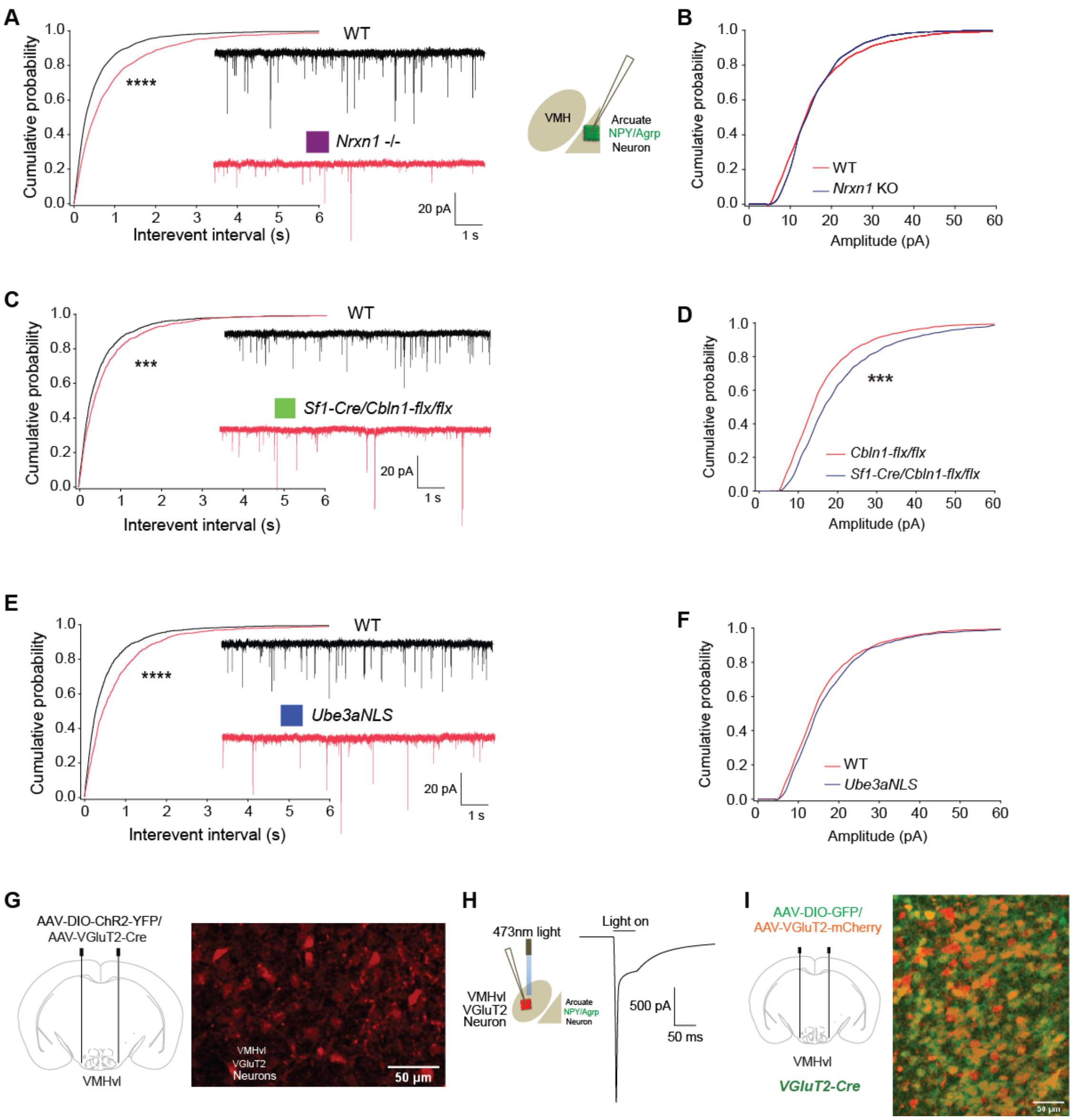

fig. S6 Miniature excitatory postsynaptic current (mEPSC) inter-event intervals and amplitude in arcuate NPY-GFP neurons of mice with deletion of *Nrxn1*, *Cbln1* or *Ube3aNLS7-1x*. (**A**) Left: Cumulative frequency plot of mEPSC inter-event intervals in arcuate NPY GFP$^+$ neurons in mice with homozygous deletion of *Nrxn1* (n = 6 mice, 19 cells) and control

bioRxiv preprint doi: https://doi.org/10.1101/2023.02.28.530462; this version posted March 1, 2023. The copyright holder for this preprint (which was not certified by peer review) is the author/funder, who has granted bioRxiv a license to display the preprint in perpetuity. It is made available under aCC-BY 4.0 International license.

mice (n = 5 mice, 14 cells, KS test, P < 0.00001); Right: diagram of whole–cell patch-clamp recordings were performed in GFP-labeled arcuate NPY/AgRP neurons. (**B**) Cumulative frequency plot of mEPSC amplitude in arcuate NPY GFP$^+$ neurons (labeled by *NPY-hrGFP* allele) in mice with homozygous deletion of *Nrxn1* (n = 6 mice, 19 cells) and control mice (n = 5 mice, 14 cells, KS test, P > 0.05). (**C**) mEPSC inter-event intervals in arcuate NPY GFP$^+$ neurons from *Sf1-Cre*:*Cbln1-flx/flx* mice (n = 5 mice, 19 cells) and control mice (n = 5 mice, 14 cells, KS test, P < 0.001). (**D**) Cumulative frequency plot of mEPSC amplitude in arcuate NPY GFP$^+$ neurons in mice with *Sf1-Cre*:*Cbln1*$^{flx/flx}$ (n = 5 mice, 19 cells) and control mice (n = 5 mice, 14 cells, KS test, P < 0.001). (**E**) mEPSC inter-event intervals in arcuate NPY GFP$^+$ neurons in mice with *Ube3a-NLS7-1x* (n = 5 mice, 17 cells) and control mice (n = 5 mice, 14 cells, KS test, P < 0.00001). (**F**) Cumulative frequency plot of mEPSC amplitude in arcuate NPY GFP$^+$ neurons in mice with *Ube3aNLS7-1x* (n = 5 mice, 17 cells) and control mice (n = 5 mice, 14 cells, KS test, P > 0.05). (**G**) Diagram of injection of AAV-hSyn-DIO-ChR2- mCherry plus AAV-VGlut2-Cre-2A-mCherry into VMHvl and representative image of VMHvl neurons expressing *mCherry.* (**H**) Diagram of whole–cell patch-clamp recordings performed in mCherry-labeled VMHvl neuron and representative trace of light-evoked ChR2 currents recorded in a VMHvl neuron. (**I**) Diagram of AAV-hSyn-DIO-GFP plus AAV-VGlut2-mCherry injected into VMHvl of *VGluT2-Cre* mice and representative image of VMHvl glutamate neurons showing overlapping expression of GFP and mCherry. Statistical significance of inter-event intervals and amplitude of mEPSC was determined by Kolmogorov-Smirnov (KS) test. P < 0.05 was considered statistically significant with *ns* indicating non-significant, *P <0.05, **P < 0.01, ***P <0.001 and ****P <0.0001.
